# Gender Differences in the Predictive Efficacy of Mayo‐PACC for Alzheimer's Disease Biomarkers: Results from a South American Cohort

**DOI:** 10.1002/alz.091506

**Published:** 2025-01-09

**Authors:** Greta Keller, Patricio Chrem Mendez, Nicolas Corvalan, Giulia Solange Clas, María Florencia Álvarez, Lucia Pertierra, Ricardo Allegri, Gustavo Sevlever, Lucia Crivelli, Ezequiel Ignacio Surace

**Affiliations:** ^1^ Fleni, Buenos Aires, CABA Argentina; ^2^ Fleni, Buenos Aires Argentina; ^3^ Fleni, Buenos Aires, Buenos Aires Argentina

## Abstract

**Background:**

With the increase in life expectancy and the rising prevalence of Alzheimer's Disease (AD), the integration of biomarkers for early diagnosis is crucial. The Mayo Preclinical Alzheimer's Cognitive Composite (Mayo‐PACC), encompassing the Rey‐Auditory Verbal Learning Test (RAVLT), Trail Making Test ‐ B (TMT B), and semantic fluency, is designed to detect cognitive changes in preclinical AD. This study investigates gender‐based differences in the predictive efficacy of Mayo‐PACC for AD biomarkers following the ATN (Amyloid, Tau, Neurodegeneration) criteria.

**Method:**

The study included 112 patients diagnosed with Mild Cognitive Impairment (MCI), comprising 38% women and 61% men, matched by age (mean ± SD: 68.73 ± 10.70 years), education (14.21 ± 3.56 years), and cognitive performance (Mayo‐PACC p= 0.76). Neuropsychological assessments were conducted using the Mayo‐PACC, and cerebrospinal fluid biomarkers (amyloid β1–42, total tau, and phosphorylated tau at threonine 181) were quantified by ELISA (Fujirebio, Japan). Biomarkers were dichotomized using cutoff values previously determined in our laboratory.

**Result:**

Random Forest models indicated an overall area under the curve (AUC) of 0.67 for the Mayo‐PACC. In gender‐specific analyses, women showed a lower predictive capacity (AUC: 0.43), with TMT B (35.42%), RAVLT (32.84%), and semantic fluency (31.74%) as key predictors. For men, a higher AUC of 0.90 was observed, with TMT B (35.49%) and semantic fluency (34.91%) being the most influential. When examining individual ATN biomarkers, women showed an AUC of 0.25 for Aβ1‐42, 0.54 for N, and 0.40 for T, with RAVLT (40.51%) as a prominent feature. Men exhibited AUCs of 0.63 for Aβ1‐42, 0.83 for N, and 0.48 for T, with semantic fluency (33.95%), and RAVLT (33.12%) being significant predictors.

**Conclusion:**

The study highlights notable gender differences in the predictive performance of neuropsychological composites for AD biomarkers. Biomarker prediction was more accurate in men. These findings suggest the importance of gender‐specific approaches in predictive modeling for AD diagnosis, underscoring the potential to enhance diagnostic precision in clinical practice.

